# Fluorescence-Guided Laparoscopy after Oral Hypericin Administration for Staging of Locally Advanced Gastric Cancer—A Pilot Study

**DOI:** 10.3390/jcm13082422

**Published:** 2024-04-21

**Authors:** Can Yurttas, Philipp Horvath, Imma Fischer, Silvia Wagner, Karolin Thiel, Ruth Ladurner, Ingmar Königsrainer, Alfred Königsrainer, Matthias Schwab, Stefan Beckert, Markus W. Löffler

**Affiliations:** 1 Department of General, Visceral and Transplant Surgery, University Hospital Tübingen, Hoppe-Seyler-Str. 3, 72076 Tübingen, Germany; 2 Department of General, Visceral and Thoracic Surgery, Landeskrankenhaus Feldkirch, Carinagasse 47, 6807 Feldkirch, Austria; 3 Institute for Clinical Epidemiology and Applied Biometry, University Hospital Tübingen, Silcherstr. 5, 72076 Tübingen, Germany; 4 Department of General, Visceral, and Thoracic Surgery, Oberschwabenklinik, St. Elisabethen-Klinikum, Elisabethenstr. 15, 88212 Ravensburg, Germany; 5 German Cancer Consortium (DKTK) and German Cancer Research Center (DKFZ), Partner Site Tübingen, 72076 Tübingen, Germany; 6 Department of Clinical Pharmacology, University Hospital Tübingen, Auf der Morgenstelle 8, 72076 Tübingen, Germany; 7 Dr. Margarete Fischer-Bosch-Institute of Clinical Pharmacology, Auerbachstr. 112, 70376 Stuttgart, Germany; 8 Departments of Pharmacy and Biochemistry, University of Tübingen, Auf der Morgenstelle 8, 72076 Tübingen, Germany; 9 Department of General and Visceral Surgery, Schwarzwald-Baar Klinikum, Klinikstr. 11, 78052 Villingen-Schwenningen, Germany; 10 Institute for Immunology, University of Tübingen, Auf der Morgenstelle 15, 72076 Tübingen, Germany

**Keywords:** laparoscopic staging, fluorescence imaging, St. John’s wort, *Hypericum perforatum*, peritoneal metastasis, peritoneal carcinomatosis

## Abstract

(1) **Background:** Laparoscopic staging is essential in gastric cancer (GC) to rule out peritoneal metastasis (PM). Hypericin, a plant-derived fluorescent compound, has been suggested to improve laparoscopic visualization of PM from GC. This prospective, single-arm, open-label clinical trial aimed to assess the feasibility and safety of oral hypericin administration as well as the suitability of fluorescence-guided laparoscopy (FGL) for improving the sensitivity and specificity of staging in GC patients (EudraCT-Number: 2015-005277-21; clinicaltrials.gov identifier: NCT-02840331). (2) **Methods:** GC patients received Laif^®^ 900, an approved hypericin-containing phytopharmaceutical, once orally two to four hours before white light and ultraviolet light laparoscopy. The peritoneal cancer index was evaluated, biopsies taken and hypericin concentrations in serum and peritoneal tissue were determined by mass spectrometry. (3) **Results:** Between 2017 and 2021, out of 63 patients screened for eligibility, 50 patients were enrolled and treated per protocol. The study intervention was shown to be feasible and safe in all patients. Standard laparoscopy revealed suspicious lesions in 27 patients (54%), among whom 16 (59%) were diagnosed with PM. FGL identified suspicious areas in 25 patients (50%), among whom PM was confirmed in 13 cases (52%). Although hypericin concentrations in serum reached up to 5.64 ng/mL, no hypericin was detectable in peritoneal tissue biopsies. (4) **Conclusions:** FGL in patients with GC was shown to be feasible but futile in this study. Sufficient levels of hypericin should be ensured in target tissue prior to reassessing FGL with hypericin.

## 1. Introduction

Peritoneal metastasis (PM) in gastric cancer is associated with poor prognosis, and prospects are bleak with 5.4 months in median overall survival [1]. Given that 27% of gastric cancer patients are diagnosed with synchronous PM at initial diagnosis [2] and computed tomography (CT) shows only limited diagnostic sensitivity for PM [3], international guidelines recommend laparoscopy for the staging of patients with locally advanced gastric cancers [4]. The sensitivity and specificity of white light laparoscopy (WLL), representing the diagnostic standard in staging PM, have been shown to be at 84% and 100 %, respectively [5]. However, visual assessment during laparoscopy is not always unequivocal, and biopsies from suspicious lesions, e.g., in the intestine, involve a high risk of perforation. Aiming to improve the sensitivity of laparoscopy and to better discriminate malignant from benign lesions, fluorescence-guided laparoscopy (FGL) has been suggested.

The main principle of FGL in cancer is that a fluorescent agent accumulates in malignant lesions and thereby enhances contrast between healthy and malignant tissue following excitation by light with the appropriate wavelength [6]. Respective approaches are already used in various malignancies, such as cancers affecting the brain, liver and the bladder [7,8,9,10,11]. Agents most commonly employed include indocyanine green (ICG) and delta aminolaevulinic acid (dALA).

Hypericin, a red fluorescent anthraquinone derivative and component of *Hypericum perforatum*, is approved for the treatment of mild depressions and has been shown to accumulate within numerous malignancies, such as gastric, lung, ovarian, bladder and colon cancer, squamous cell carcinoma, glioblastoma, rhabdomyosarcoma, fibrosarcoma and nasopharyngeal carcinoma [12,13,14,15,16,17,18,19,20]. Its photoactive properties result in an increased risk of photodermatitis after oral intake [21,22,23] and constitute a common side-effect of *Hypericum perforatum* administration. Concurrently, these properties make it suitable for FGL [21,24] and may allow for additional photodynamic therapy inducing autophagy, apoptosis, necrosis and immunogenic cell death [25,26,27,28,29,30], due to the formation of reactive oxygen species (ROS) following irradiation [31]. Cytotoxic effects connected with hypericin are oxygen-dependent [32], and apoptosis induction or necrosis has been demonstrated to be dependent on drug concentrations and the duration of exposure to irradiation [33]. Since light penetration into tissues is limited, phototoxic effects remain shallow [34,35]; therefore, photodynamic therapy with hypericin has mainly been applied to superficial tumor lesions in skin [36,37,38] and bladder cancers [39,40,41]. Further, in an abdominal rhabdomyosarcoma mouse model, improved intraperitoneal tumor visualization with hypericin was established and the simultaneous effects of photodynamic therapy verified [42].

The objective of this present clinical study was therefore to investigate whether FGL is feasible after oral Laif^®^ 900 (*hypericum perforatum*) administration and suited to improving the detection of PM when staging patients with locally advanced gastric adenocarcinomas. Moreover, we intended to assess the pharmacokinetics of hypericin in serum, as well as in biopsies from benign and malignant peritoneal tissue, and to investigate a potential application of hypericin for photodynamic therapy of PM.

## 2. Materials and Methods

### 2.1. Trial Design

The primary objective of this present clinical study was to assess whether FGL with hypericin is able to improve the detection of PM in the staging of patients with locally advanced gastric adenocarcinomas compared to standard WLL alone. The detection of PM in locally advanced gastric adenocarcinomas was compared between standard WLL and FGL with hypericin. If suspect lesions were present, their tumor size and intra-abdominal localization were also assessed. As a secondary objective, hypericin levels were to be assessed both in blood as well as in healthy-appearing and malignant tissue samples. This study was set up in two stages: In the first stage, patients were enrolled regardless of whether PM was suspected in preoperative staging CT scans or not. This stage was planned to be terminated after ≥20 patients had been enrolled in the trial and as soon as 8 patients with CT-morphological signs of PM were among them (both conditions apply). The second stage was planned to be initiated only after establishing that the feasibility of FGL could be shown in the first stage, i.e., that the planned procedure was shown to be feasible in ≥75% of patients included at this stage (e.g., feasibility in ≥15 of the 20 patients included) and fluorescent lesions can be determined by FGL in ≥50% of the patient subgroup showing CT-morphological signs of PM. At the second stage, only patients without CT-morphological signs of PM were eligible for enrollment, up to a total of 50 patients.

### 2.2. Drug Treatment

Patients received Laif^®^ 900 (Bayer AG, Leverkusen, Germany), a dry ethanol extract (80% (vol.%); 900 mg) obtained from *Hypericum perforatum* and formulated as a film-coated tablet, containing hypericin per os two to four hours prior to staging laparoscopy.

### 2.3. Inclusion/Exclusion Criteria

Inclusion criteria comprised patients aged ≥ 18 years, with adequate performance status (Karnofsky performance score > 70), willing to use a safe method of contraception with histologically confirmed gastric adenocarcinomas and CT-morphological signs of locally advanced malignancies (≥cT3 and/or cN+). Exclusion criteria included suspected distant metastasis except for PM, patients considered inoperable due to comorbidities or general health status, pregnancy or breast-feeding, patients with contraindications against the study medication (e.g., known allergies) as well as patients unable or unwilling to give their informed consent. Further patients suffering from secondary malignancies or participating in other interventional trials were excluded from study enrolment.

### 2.4. Trial Approval and Consent to Participate

This trial was conducted in accordance with applicable laws and regulations as well as the principles of the Declaration of Helsinki. Clinical trial approval was received by the local institutional review board (Ethikkommission an der Medizinischen Fakultät der Eberhard-Karls-Universität und am Universitätsklinikum Tübingen, Project No. 803/2015AMG1 on 7th of March 2016) and the German Federal regulatory authorities (Federal Institute for Drugs and Medical Devices—Bundesinstitut für Arzneimittel und Medizinprodukte; BfArM). Informed consent for study participation and subsequent publication of study results was obtained from all enrolled patients and documented in writing, with full understanding of the experimental procedures prior to any study interventions. The trial was registered with the European Union Drug Regulating Authorities Clinical Trials Database under EudraCT-number: 2015-005277-21 and with clinicaltrials.gov under the identifier NCT-02840331, which have been completed. Where applicable, this trial conforms with guidelines of the International Committee of Medical Journal Editors (ICMJE) and Consolidated Standards of Reporting Trials (CONSORT).

### 2.5. Study Conduct

Gastric cancer patients of both sexes were screened for eligibility when presenting at our outpatient clinic. All patient cases were discussed by a multidisciplinary tumor board that recommended further treatment and study enrollment. Patients were enrolled according to the inclusion/exclusion criteria mentioned above, and written informed patient consent was unequivocally required. Patients fasted before hospital admission starting at 6:30 a.m. prior to laparoscopy. Study medication (Laif^®^ 900) was ingested under the direct supervision of a study investigator.

### 2.6. Laparoscopy

All surgical procedures were performed under general anesthesia. When indicated, a port-catheter was also implanted during the intervention. First, the abdominal cavity was accessed via the introduction of a Veress needle and CO_2_ insufflated. A 12 mm optic trocar was introduced under optical control with a 30° camera (Karl STORZ SE & Co. KG, Tuttlingen, Germany). One or two additional 5 mm trocars were placed at the surgeon’s discretion. The abdominal cavity with its 13 abdominopelvic regions was systematically inspected for lesions suspicious for PM consecutively using WLL and FGL and assessed according to the PCI method described by Jacquet et al. [43]. When necessary, intra-abdominal adhesions were removed for improved sight. Peritoneal washing cytology was performed by flushing the abdominal cavity with 250 mL of saline and reabsorbing it or ascites used when abundantly present. FGL with blue light at a wavelength of 390–440 nm was performed using the OPAL1™ technology (Karl STORZ SE & Co. KG, Tuttlingen, Germany) in a time window from two to four hours following the ingestion of study medication. Tissue for histopathological assessment and hypericin determination was sampled from the parietal peritoneum appearing as benign and, if present, PM-suspicious lesions during both WLL and FGL procedures, respectively. In the case of suspected PM, selected peritoneal lesions were UV-irradiated for 15 min at a location easily accessible for sampling.

### 2.7. Determination of Hypericin

Tissue samples and serum obtained from study patients was analyzed by high-performance liquid chromatography (HPLC) tandem mass spectrometry (MS/MS) using a validated method (Prolytic GmbH, Kymos Group, Frankfurt am Main, Germany). Quality control samples of human serum spiked with hypericin at defined concentrations were used to ensure the validity of the technique. Calibration curves and quality control samples of spiked serum samples with defined hypericin concentrations were established over a range from 0.2 to 20 ng/mL.

### 2.8. Statistical Analysis and Data Availability

To analyze the primary outcome criterion, the frequency of visual identification of PM suspect lesions among locally advanced adenocarcinoma patients detected by FGL after hypericin administration is compared with the respective frequency by standard WLL (gold standard) based on the results of the intention-to-treat (ITT) population. The ITT population includes all patients for whom both the standard WLL and FGL were planned, whereas the per protocol (PP) population includes all patients of the ITT population for whom an FGL was successfully performed and who had no serious protocol violations. The identification of PM suspect lesions is operationalized by the peritoneal cancer index (PCI), which is documented after WLL as well as after WLL combined with FGL. If no PM suspect lesions are identified, the PCI value equaled “0”; if PM is suspected, the resulting PCI was “>0”. The comparison of results from the two diagnostic procedures (WLL and FGL) is based on the PP population by determining sensitivity and specificity and positive (PPV) and negative (NPV) predictive value for FGL with hypericin, using the outcomes of WLL as the gold standard. For the secondary outcome variables, the concentration of hypericin in unaffected peritoneal tissue and tumor tissue as well as in patient serum was determined.

In descriptive analyses, the ITT and PP populations are described with respect to all documented parameters. The distributions of continuous parameters are presented in tabular form with case numbers, means, standard deviation, quartiles and extremes. The distributions of nominally or ordinally scaled parameters are characterized by contingency tables in which the absolute and relative frequencies are given. All statistical analyses were performed with SAS version 9.4 (SAS Institute GmbH, Heidelberg, Germany). The datasets used and analyzed during this study are available from the corresponding author on request.

Data are stored in controlled access data storage at the University Hospital of Tübingen, Germany. For patient data safety reasons, data are not openly accessible but may be requested from the corresponding author upon reasonable explanation.

### 2.9. Safety Analysis

The percentage of patients with (severe) adverse events (AEs/SAEs) according to the Common Terminology Criteria for Adverse Events (CTCAE) version 4.0 documented after staging laparoscopy during their hospital stay was assessed as the outcome measures for the safety analysis.

## 3. Results

### 3.1. Study Enrollment and Patient Characteristics

**Figure 1:**
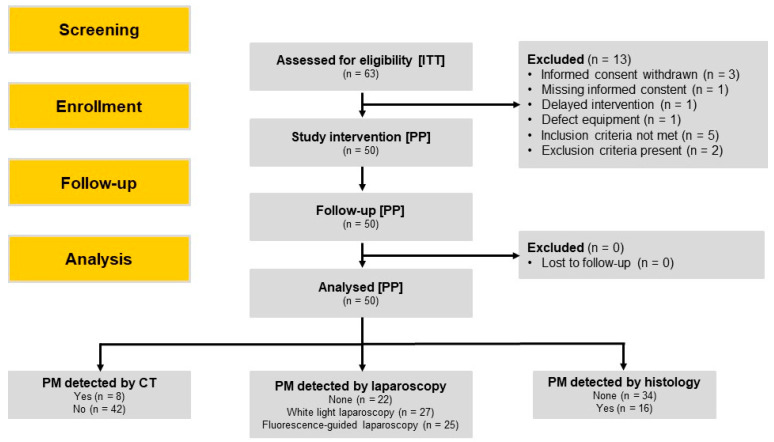
Screening, enrollment, follow-up, analysis and results of patients with locally advanced gastric cancer that were scheduled for staging laparoscopy (the layout was adapted from the CONSORT 2010 statement [44]). ITT—intention to treat; PP—per protocol population.

Overall, 63 patients with gastric adenocarcinomas were screened for study eligibility, among whom 50 patients were included in the trial and allocated to study intervention, thus comprising the ITT population. All enrolled patients were at a locally advanced tumor stage (cT ≥ 3 and/or cN+) without clinical or radiological signs of extraperitoneal metastases, for whom staging laparoscopy is recommended in potentially resectable cases according to the European Society for Medical Oncology (ESMO) clinical practice guidelines for gastric cancer [4]. Characteristics of patients treated per protocol and their cancers are provided in Table 1. Of those excluded, eight patients (12.7%) did not meet the inclusion/exclusion criteria, yet one patient was included according to the investigator’s assessment in spite of exclusion criteria applying with minor deviations in renal function (glomerular filtration rate [GFR]: 57.5 mL/min/1.73 m^2^; limit: ≥60 mL/min/1.73 m^2^). Three patients withdrew their informed consent and were therefore excluded. Among the other three patients that were considered screening failures, one patient each was excluded due to missing informed consent, delayed study intervention following administration of study medication and malfunctioning study equipment. A flow diagram of the trial including screening and enrollment of patients is depicted in Figure 1.

### 3.2. Assessment of PM by CT, White Light (WLL) and Fluorescence-Guided Laparoscopy (FGL)

**Figure 2:**
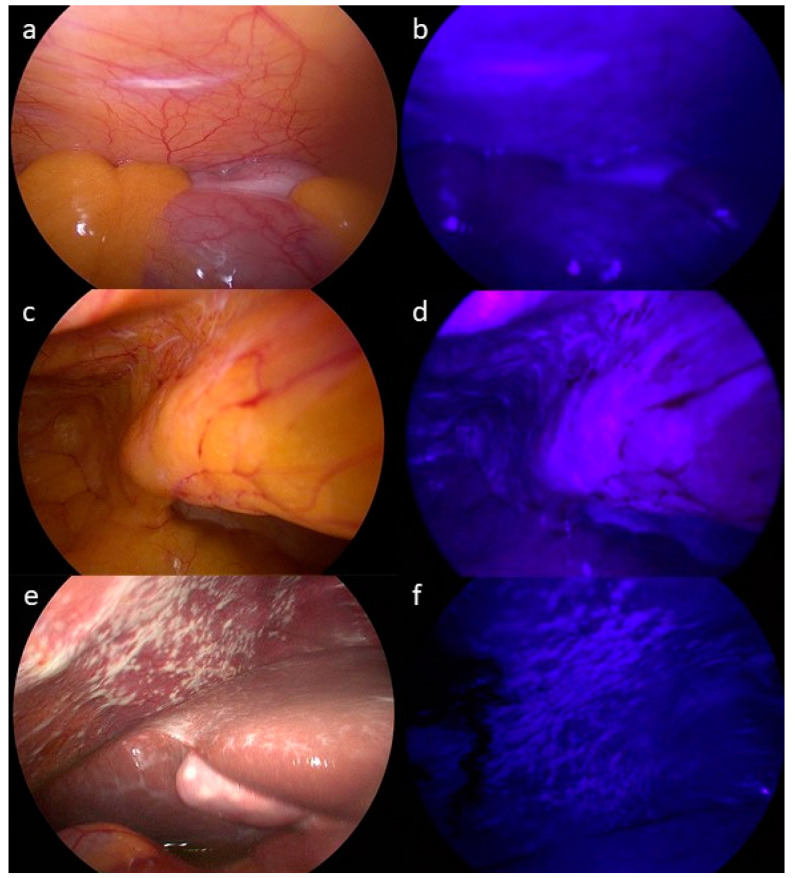
(**a**–**f**): Exemplary intraoperative macroscopic views of patients during white light laparoscopy (WLL; **left**) and corresponding images from fluorescence-guided laparoscopy (FGL; **right**). (**a**,**e**) show macroscopic signs of PM, whereas (**c**) depicts an example of unsuspicious peritoneal tissue identified by WLL. (**b**,**d**) represent examples of fluorescent areas discernible during FGL, whereas (**f**) lacks discernible fluorescent areas. Histopathological examination confirmed PM in the lesions shown in (**a**,**b**,**e**,**f**), whereas unspecific inflammation was determined in biopsies from (**c**) (unsuspicious peritoneal tissue) and (**d**) (fluorescent area).

**Figure 3:**
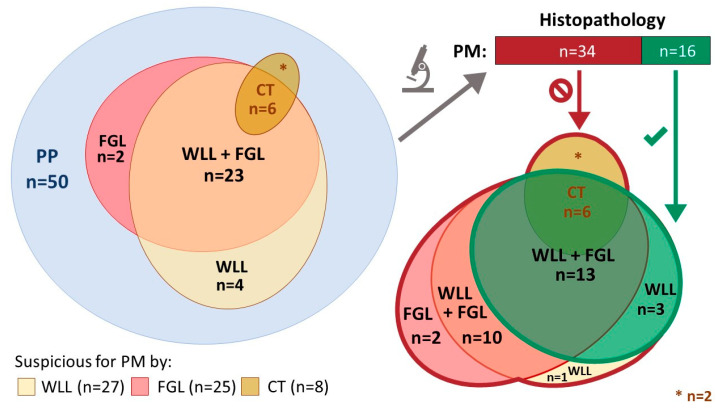
Venn diagram illustrating the diagnostic results of patients that were treated per protocol (PP). Out of eight patients with indications of peritoneal metastasis (PM) identified by CT, six patients had signs of PM both during white light laparoscopy (WLL) and fluorescence-guided laparoscopy (FGL), and two patients remained negative. With WLL, PM was suspected in 27 patients, whereas FGL showed lesions suspicious for PM in 25 patients—in 23 patients, lesions were found to be suspicious by both WLL and FGL. In four patients with suspicious peritoneal lesions during WLL, no fluorescence was detectable during FGL. In two patients with fluorescent lesions identified by FGL, no lesions were found during WLL. In these two patients, histology showed unspecific chronic inflammation but no malignancy. In total, histopathological assessment proved positive (green arrow and border) for PM in 16 patients (32%), while findings in biopsies from 34 patients (68%) remained negative (red arrow and borders). During WLL, all 16 patients confirmed to be positive for PM via histopathology were identified, compared to only 13 patients during FGL. False-positive findings were obtained in ten patients simultaneously by both WLL and FGL, while WLL and FGL misidentified lesions in one and two patients, respectively. When benchmarked against histopathology, CT imaging correctly identified PM in six cases, while in two patients, no malignancy could be determined in biopsies with histopathology. FGL—fluorescence-guided laparoscopy; PM—peritoneal metastasis; PP—per protocol population; WLL—white light laparoscopy. The tile size used for diagrams is arbitrary.

In 6 out of the 8 patients (75%) who showed CT-morphological signs of PM prior to laparoscopy enrolled in stage 1 of this study, suspect lesions could be identified with both standard WLL and FGL. Histopathological examination confirmed PM in all 6 of those patients. Among the 42 patients additionally enrolled during stage 2 of the trial, there were 21 patients (50%) that showed suspicious lesions consistent with PM during standard WLL, among whom diagnoses were confirmed by histopathological examination of biopsies in 10 cases (24%). Thus, altogether, among 27 patients in whom macroscopic lesions suspicious for PM were identified by WLL, diagnoses were confirmed by histopathological assessment of biopsies in 16 cases (59.3%). FGL with hypericin showed fluorescent lesions optically consistent with PM in 25 patients (50%). Histology confirmed PM in 13 of those 25 patients (52%). In three patients, no fluorescence was observed despite macroscopic signs consistent with PM in WLL and subsequent histopathological confirmation. In two patients (4%), PM was suspected during FGL, but WLL did not show any suspicious lesions, and histology did not prove the presence of PM. Two out of three patients with signs of PM during WLL and who showed no fluorescence during FGL were confirmed as having PM via histology. Examples of intraoperative laparoscopic views are shown in Figure 2. The results are depicted by Venn diagram in Figure 3.

### 3.3. Accuracy of CT, WLL and FGL in Detecting PM

In this study, the presence of PM was confirmed by histopathological examination of biopsies taken during laparoscopy. However, as a limitation, it should be noted that many suspicious lesions within the abdominal cavity are inaccessible for biopsy and therefore also evade histopathological examination, which mainly applies to PM affecting the visceral peritoneum of the intestine or locations where the peritoneum covers major blood vessels. Moreover, not every suspicious lesion that is discernible during laparoscopy can be sampled and examined using histology. Compared with WLL, FGL with hypericin yielded a sensitivity of 85.2% (95% CI 71.8–98.6) and a specificity of 91.3% (95% CI 79.8–100). When determining the sensitivity and specificity of WLL and FGL on the basis of histopathological assessments as the gold standard, the sensitivity of WLL in detecting PM was 100% and the specificity was 67.6% in our cohort treated per protocol (PP). In contrast, FGL yielded a sensitivity of 81.3% and a specificity of 64.7%. Sensitivity and specificity of CT, WLL and FGL are summarized in Table 2.

### 3.4. Safety Assessment

Safety was assessed for all patients who received study medication (*n* = 53) until the end of the study on day one after laparoscopy. In two patients, one adverse event (AE) each was recorded. During laryngoscopy for orotracheal intubation, a suspicious oral lesion was identified and inspected by a consultant of otolaryngology and assessed as a benign lesion. The other recorded AE consisted of urinary retention following urinary catheter removal following surgery and required catheter reinsertion and oral tamsulosin administration in a patient with benign prostatic hyperplasia. Both of these AEs were considered unrelated to the study treatment. Hence, two patients (3.8%) out of the fifty-three patients dosed in total experienced postoperative AEs during the study. Safety surveillance was terminated when patients were discharged from hospital.

### 3.5. Determination of Hypericin in Serum and Peritoneal Tissue Samples

**Figure 4:**
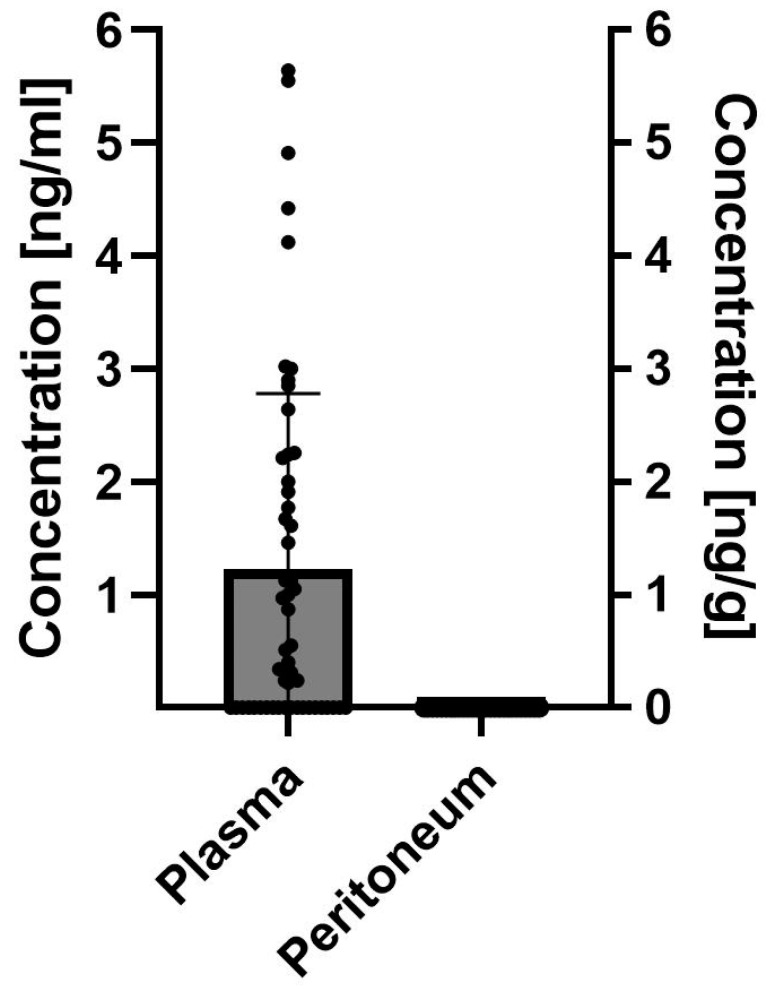
Hypericin concentration as assessed by HPLC MS/MS in serum and peritoneal tissue samples of study patients who had received Laif^®^ 900 two to four hours prior to sampling. Lower limit of quantification (LLOQ) for serum: 0.02 ng/mL; LLOQ for tissue: 1 ng/g tissue; single dots represent measured values for each patient.

In total, 77 study samples were assessed for hypericin concentrations by HPLC–MS/MS (50 serum samples and 27 peritoneum biopsy samples). Hypericin could be quantified (above the lower limit of quantification (LLOQ) of 0.2 ng/mL) in serum samples obtained from 30 patients (60.0%), whereas in 20 patients, the concentrations remained below the LLOQ threshold. Respective hypericin concentrations in serum ranged between 0.22 and 5.64 ng/mL with a mean concentration of 1.97 ng/mL (±1.61) among the patients with detectable hypericin serum levels (*n* = 30). In the 27 peritoneum samples obtained from the 26 patients available and considered suitable for assessment of hypericin, none showed quantifiable concentrations of hypericin (LLOQ of 1 ng/g tissue). Samples were taken from peritoneal tissue that was considered unaffected by PM during WLL and FGL in 17 cases (63.0%). Two samples (7.4%) were considered as affected by PM in WLL only, five (18.5%) as affected by PM during FGL only and three macroscopically assessed as PM (11.1%) by both WLL and FGL. The results are depicted in Figure 4.

### 3.6. Photodynamic Therapy

Considering that no hypericin was detectable in peritoneal tissue samples from any of the patients who had received Laif^®^ 900 in our clinical study, we refrained from the determination of possible cytotoxic effects potentially induced through the intended photodynamic therapy in PM-suspicious peritoneal lesions that had been exposed to ultraviolet light for 15 min during laparoscopy, although this had initially been foreseen in the study protocol.

## 4. Discussion

During this study, we aimed to assess the feasibility as well as the sensitivity and specificity of FGL following the oral administration of hypericin containing Laif^®^ in patients with locally advanced gastric adenocarcinoma. Mass spectrometry established detectable amounts of hypericin in serum samples obtained 2–4 h following oral Laif^®^ intake in the majority of dosed patients. Nevertheless, in peritoneal samples, neither benign nor tumor-affected tissue contained hypericin in quantifiable amounts. Due to this outcome, we refrained from further investigations of apoptosis induction in irradiated tissue as initially planned. While the technical feasibility of conducting FGL could be established, neither improved sensitivity nor specificity could be shown as compared to WLL. Accordingly, this study should be considered a negative trial since the primary objective (improved detection of PM from gastric cancer by FGL) has not been met. It stands to reason that this finding might be due to insufficient drug concentrations in the target tissue, and further, uptake into the peritoneum after oral administration may be limited. However, in a substantial proportion of patients, serum levels of hypericin were measurable at the time of laparoscopy, suggesting that in general oral ingestion of *Hypericum perforatum* may be a suitable method of drug administration.

Of note, a preclinical model with intraperitoneal rhabdomyosarcoma-bearing mice showed that FGL with intravenously administered hypericin can improve the discrimination of tumor and benign tissue, whereas in this study, no fluorescence could be observed following ICG injection [42]. Although neither serum nor tissue concentrations were determined for hypericin in this animal model, intravenous administration of hypericin can ensure more consistent serum concentrations and may circumvent possible issues with drug liberation, absorption, distribution, metabolism and excretion (LADME) experienced with oral intake. Moreover, injections of hypericin would allow for a more defined or even body-weight-adapted dosing of the active substance hypericin, whereas uniform oral doses of *Hypericum perforatum* may contain differing amounts of hypericin and thereby contribute to varying serum levels. However, to date, there is no approved hypericin-containing drug available for intravenous application in humans.

Regarding pharmacokinetics, the findings from the blood samples of 12 male patients after oral intake of dried *Hypericum perforatum* extract containing both hypericin and pseudohypericin have suggested an absorption lag time of 2.0–2.6 h for hypericin compared to 0.3–1.1 h for pseudohypericin [45], guiding the design of our study. Assuming that hypericin has locally accumulated, photostability for up to 16 h with lasting fluorescence has been demonstrated in tumor tissue [46]. Nevertheless, considering the limited bioavailability of the hydrophobic hypericin with yet unknown pharmacokinetics concerning LADME and peritoneal deposition after oral intake, an earlier ingestion of *Hypericum perforatum* before laparoscopy or an intravenous or topical drug application during laparoscopy might be preferable and prove more effective in obtaining the desired fluorescence signals. Of note, the pharmaceutical drug information (summary of product characteristics) of Laif^®^ 900, which was available during the conceptualization of the trial until 2019, stated that hypericin uptake peaks two hours after oral intake, while the revised version, available since 2020, includes additional pharmacokinetic characteristics: the maximum plasma level of hypericin is reported to peak at 3.8 ± 1.4 ng/mL after 7.9 ± 1.3 h, whereas pseudohypericin achieves maximum plasma levels at 10.2 ± 3.9 ng/mL after 2.7 ± 0.7 h. Therefore, this may be among the underlying reasons for the insufficient tissue concentrations of hypericin observed two–four hours following oral intake in our study.

With different pharmaceuticals, e.g., with orally administered dALA, FGL performed better when aiming for the identification of intra-abdominal tumors and/or metastasis in gastric cancer patients, since tumor-affected areas were better discernible as compared to standard WLL [47]. Nevertheless, in this context, inflamed tissue proved to be an obstacle due to fluorescence induction, thereby producing false-positive findings, which was also observed in our study cohort. Therefore, we have likely identified inflamed and other areas with autofluorescence during our study with FGL for biopsy. Orally administered dALA was also investigated for intraoperative visualization of breast cancer and lymph node metastases in a small cohort of 16 patients. Although tumor tissue showed increased fluorescence compared to the surrounding tissue, discrimination of tumor-affected vs. unaffected lymph nodes could not be improved through fluorescence [48]. In contrast, in non-muscle invasive bladder cancer, fluorescence-guided transurethral resection following oral dALA administration improved surgical results compared to standard white light cystoscopic resection, when regarding residual tumors and short-term local recurrences [49,50,51]. Further, improved sensitivity through oral dALA could be shown in a clinical trial of staging advanced gastric adenocarcinoma patients using laparoscopy. Here, 52 patients received dALA 3 to 4 h prior to laparoscopy, and findings consistent with PM were observed in 28 patients using standard WLL, among whom 24 were diagnosed with PM using histopathology. However, among the remainder of the 24 patients considered to be without PM using WLL, another 5 patients were identified by FGL, and PM was confirmed in biopsies using histopathology. On the downside, there were false-negative cases with histopathologically confirmed PM that went undetected by FGL, as well as false-positives caused by inflammatory processes [52].

Taken together, FGL seems technically feasible after oral administration of a phytopharmacon containing a fluorescent drug. Regarding hypericin, the determination of serum and tissue levels from patients after one single oral administration of Laif^®^ unfortunately resulted in comparably low [45] or no detectable hypericin serum concentrations and no measurable hypericin in tissue biopsies in our study. Therefore, it is likely that a different interval between oral intake and laparoscopy or a higher dosage of hypericin might achieve improved serum and tissue levels to allow for target-specific fluorescence and lead to potential benefits through FGL. Notably, *Hypericum perforatum* contains varying amounts of hypericin, and the compounds contained in it also interact with cytochrome P450 enzymes, which makes precise calculations and predictions of serum pharmacokinetics difficult [53]. Another potential method of hypericin administration would obviously be intravenous or even local application into the peritoneal cavity, which might circumvent pharmacokinetic issues. The feasibility of intra-abdominal application of hypericin to enhance tumor detection has been demonstrated recently in an animal model of peritoneal metastasis [54]. Nevertheless, hypericin is not an approved drug for intravenous or intra-abdominal use and is so far unavailable.

The most important limitation of our study is likely the study conduct, without ensuring in advance that sufficient concentrations of hypericin can be reached in the peritoneum and the target tissue. Fluorescence observed during laparoscopy was likely unspecific, while hypericin may speculatively possess fluorescent properties in tissue even below the lowest level of quantification, which was at 1 ng/g for our trial. Another limitation of this study is certainly that only patients with gastric adenocarcinoma were included, of whom 10 (20%) received neoadjuvant chemotherapy. Prior systemic treatment might have affected hypericin uptake into PM. At this point, we are also unable to state if there might be any potential advantage in the laparoscopic assessment of PM with different tumor entities.

## 5. Conclusions

Taken together, our study has demonstrated the technical feasibility of FGL with hypericin in the laparoscopic staging of patients with locally advanced gastric cancer, but it failed to establish any clinical benefit in terms of sensitivity and specificity in detecting PM. Considering that no hypericin was detectable in peritoneal tissue using mass spectrometry, despite measurable serum concentrations in most of the enrolled patients, it is impossible to generally exclude any potential benefits of applying hypericin in this clinical setting. Among the lessons learned from this investigation is therefore that, prior to assessing FGL for the detection of PM with any photoactive agent, it would be advisable to first establish that sufficient tissue levels can actually be obtained with the investigated drug and route of administration.

## Figures and Tables

**Table 1:** Patient and cancer characteristics.

Patients Treated Per Protocol [PP] (*n* = 50)	
Sex—n (%)	
Female	17 (34)
Male	33 (66)
Age–Median (min–max)	
Years	62.7 (27–86)
Ethnicity—n (%)	
Caucasian	50 (100)
Location of tumor—n (%)	
Esophagogastric junction	22 (44)
Corpus ventriculi	24 (48)
Antrum ventriculi	4 (8)
Tumor extension—n (%)	
cT1	0 (0)
cT2	5 (10)
cT3	26 (52)
cT4	1 (2)
cTx	18 (36)
Nodal status—n (%)	
cN0	6 (12)
cN+	43 (86)
cNx	1 (2)
Metastasis—n (%)	
cM0	42 (84)
cM1	8 (16)
Grade of differentiation—n (%)	
G1	0 (0)
G2	14 (28)
G3	32 (64)
Gx	4 (8)
Neoadjuvant chemotherapy prior to laparoscopy—n (%)	
Yes	10 (20)
No	40 (80)

**Table 2:** Sensitivity and specificity of computed tomography, standard WLL and FGL when using hypericin to identify PM when considering histopathology assessment as the reference (gold standard).

	Sensitivity	Specificity
Computed tomography (CT)	6 of 16 (37.5%)	32 of 34 (94.1%)
White light laparoscopy (WLL)	16 of 16 (100%)	23 of 34 (67.6%)
Fluorescence-guided laparoscopy (FGL)	13 of 16 (81.3%)	22 of 34 (64.7%)

## Data Availability

The data that support the findings of this study are available on request from the corresponding author. The data are not publicly available due to privacy or ethical restrictions.
